# Exploring Barriers and Facilitators to Engagement of an Online Acceptance and Commitment Therapy Intervention for Cancer Survivors With Chronic Painful Chemotherapy-Induced Peripheral Neuropathy: Qualitative Interview Study

**DOI:** 10.2196/64983

**Published:** 2025-09-26

**Authors:** Daniëlle van de Graaf, Marije van der Lee, Tom Smeets, Hester Trompetter, Floortje Mols

**Affiliations:** 1 Tranzo, Tilburg School of Social and Behavioral Sciences, Tilburg University, Warandelaan 2, Tilburg, 5000 LE, The Netherlands, 31 13 466 4633; 2 Department of Research, Netherlands Comprehensive Cancer Organisation (IKNL), Utrecht, The Netherlands; 3 Department of Medical and Clinical Psychology, Tilburg School of Social and Behavioral Sciences, Tilburg University, The Netherlands; 4 Scientific Research Department, Centre for Psycho-Oncology, Helen Dowling Institute, Bilthoven, The Netherlands

**Keywords:** chemotherapy-induced peripheral neuropathy, cancer, online intervention, engagement, facilitators, barriers

## Abstract

**Background:**

Online self-management interventions for cancer survivors are increasingly being used, but engagement is often difficult for patients. Given the importance of engagement for intervention effectiveness, identifying patient-reported barriers and facilitators is essential.

**Objective:**

The aim of this study was to qualitatively examine barriers and facilitators influencing engagement with an online self-management intervention, offered with or without guidance, for cancer survivors experiencing chronic painful chemotherapy-induced peripheral neuropathy (CIPN).

**Methods:**

Patients who took part in the Embrace Pain randomized controlled trial, conducted between December 2021 and July 2024, were invited to participate in this study. Eligible participants were adults with chronic painful CIPN, based on criteria including pain, completion of chemotherapy, and European Organisation for Research and Treatment of Cancer QLQ-CIPN20 Questionnaire (ie, cancer-specific measure of sensory, motor, and autonomic neuropathy). The Embrace Pain randomized controlled trial involved evaluating an online self-management acceptance and commitment therapy intervention for pain interference in daily life, with some participants receiving email guidance and others not. Thereafter, 12 patients experiencing chronic painful CIPN participated in semistructured interviews. Data were analyzed using thematic analysis. An inductive coding approach was applied, and Atlas.ti (Lumivero) was used for coding.

**Results:**

In total, 2 themes and 17 codes emerged from the data, namely 7 codes for barriers and 10 codes for facilitators. Barriers included program schedule, burden, lack of guidance, irrelevance, mindfulness exercises, usability, and missing content. Facilitators included usability, recognition, positive self-management, program schedule, symptom management, relevance, guidance, experiential exercises, mindfulness exercises, and value-based living. Program schedule, guidance, mindfulness exercises, and usability proved to be barriers for some, while others indicated that they were facilitators for their use.

**Conclusions:**

Participants’ perceptions of the intervention varied, with engagement influenced by individual circumstances. These variations highlight the importance of personal context in shaping both uptake and effectiveness, indicating a need for tailored approaches to address diverse needs and challenges faced by participants.

## Introduction

The use of online interventions for patients with cancer is growing due to their benefits in costs, accessibility, availability, traveling, stigma, and psychological burden [1,2]. Previous studies into online interventions have often demonstrated positive patient outcomes [3,4], including patient groups with long-term consequences of cancer and treatment, such as patients with cancer undergoing chemotherapy-induced peripheral neuropathy (CIPN) [1]. However, studies into online health interventions raise significant issues regarding the intended use [5,6], and thus the implications for effectiveness. Research on eHealth usage is often primarily centered on adherence (ie, a quantitative behavioral approach to usage and the extent to which users benefit optimally from the intervention [7,8]). This approach neglects the qualitative dimensions of use that are critical for achieving individual health goals and therapeutic benefit [8-11]. Engagement offers a broader conceptualization, encompassing both behavioral adherence and subjective experiences such as motivation, satisfaction, and perceived relevance [8,12]. Considering engagement in online interventions is therefore crucial to maximize the effectiveness of online interventions [5,12,13].

Several studies have explored factors influencing user engagement with online interventions. One systematic review proposed an integrative framework for engagement in digital behavior change interventions, identifying 3 key determinants: intervention design (eg, content and delivery), contextual factors (eg, setting and population), and the targeted behavior [12]. Although this framework has yet to be empirically validated, it highlights the importance of considering variables that affect intervention uptake. A systematic scoping review evaluating concepts and components of engagement similarly emphasized the role of content and design in affecting engagement and adherence [8]. These considerations are particularly relevant for online self-management interventions targeting chronic symptoms, such as CIPN, where symptom management involves ongoing, complex, and time-consuming tasks [14]. This highlights the need to explore the various factors that affect user engagement in studies developing such online interventions.

In recent years, research on patients’ experiences of online self-management interventions has grown. A systematic review of both quantitative and qualitative studies identified a range of personal and psychological predictors of engagement, including the availability of guidance and sufficient time to complete the intervention [15]. The study concluded that personal and psychological factors, such as the need for guidance and sufficient time to complete the intervention, influence its use. In addition, intervention-related and technical factors, such as impersonal content and technical difficulties, were found to negatively impact user engagement [15]. Additional qualitative research has identified various facilitators and barriers to engagement. A study exploring the experiences of patients with neck and lung cancer, participating in a guided self-management intervention for psychological distress, reported both negative (eg, low levels of motivation and self-discipline) and positive experiences (eg, coaching support and engaging with assignments) [16]. However, since the intervention was offered in both print and online formats, no technology-specific insights could be derived. In contrast, another qualitative study focused exclusively on an online self-management intervention for cancer-related distress and identified 5 overarching influencing factors: illness-related, psychological, personal, intervention-related, and computer-related aspects [17]. Another study has qualitatively explored barriers and facilitators of an online mindfulness-based cognitive therapy for patients with cancer, finding factors of treatment setting, format, therapist, and patient characteristics [18]. In conclusion, these findings highlight the complex and multifaceted nature of engagement with online interventions. Previous research has demonstrated that incorporating guided elements, such as feedback, clear explanations, motivational support, and reminders, can enhance both user engagement and intervention effectiveness [19,20]. This underscores the need to understand the factors that shape patients’ engagements and experiences.

It is unclear whether previously studied influencing factors to engagement in online self-management interventions are generic. Evaluating individual interventions is crucial for optimal effectiveness. Therefore, this qualitative interview study aims to explore the barriers and facilitators influencing engagement with Embrace Pain, an online self-management intervention designed for cancer survivors experiencing chronic painful CIPN [21]. This analysis is part of a larger randomized controlled trial (RCT), assessing the effectiveness of the intervention, which is based on acceptance and commitment therapy (ACT) compared with a waiting list condition (WLC) on pain interference in daily life [22].

## Methods

### Study Design (Qualitative Study Embedded in RCT)

This qualitative study included in-depth semistructured interviews to examine the barriers and facilitators that cancer survivors with chronic painful CIPN experienced in using an online self-management intervention for cancer survivors with chronic painful CIPN, called Embrace Pain [21]. The interviews focused on individuals’ subjective experiences regarding their use of Embrace Pain.

### Positionality of Research Team

The positionality of the research team is presented in Table 1.

**Table 1.** Positionality of the research team.

Research person initials	Credentials	Occupation	Gender	Experience and training
DvdG	PhD (Medical and Clinical Psychology)	Postdoctoral researcher	Woman	Research experience on eHealth and cocreation
MvdL	PhD (Clinical Psycho-Oncology)	Endowed professor and licensed mental health psychologist	Woman	Clinical and research experience on efficacy and working mechanisms of psychological interventions in oncology, with special attention for online interventions
TS	PhD (Clinical Psychology)	Full professor	Man	Research experience on stress and trauma in relation to mental disorders
HT	PhD (Clinical Psychology)	Researcher and teacher	Woman	Research experience on psychosocial influences on quality of life, functioning, and mental health in chronic pain
FM	PhD (Medical Psychology)	Associate professor	Woman	Research experience on quality of life in cancer patients (with CIPN^a^)

a CIPN: chemotherapy-induced peripheral neuropathy.

### Overview of Embrace Pain

The online self-management intervention Embrace Pain includes psychoeducation and all ACT processes. Patients learn to perform value-oriented activities in daily life with pain, to reduce pain avoidance and increase pain acceptance. ACT is a third generation of cognitive behavioral therapy (CBT) [23]. The main therapeutic goal of ACT is to focus attention on personally valuable activities by stimulating pain acceptance, as an alternative to (experiential) avoidance. ACT has been proven effective for several types of chronic pain [24,25] and is increasingly implemented in global cancer care [26,27]. However, there had been no previous research on ACT for CIPN, although a study into cognitive and behavioral intervention for CIPN had demonstrated positive results [1]. Due to similar aspects and the greater suitability of ACT compared with CBT for the current patient population, it was expected that the current ACT-based intervention would yield positive effects [22].

An overview of the intervention of the main study is illustrated in Table 2 [21,22]. The RCT’s method, including an overview of the intervention, was previously published in a protocol paper [22] along with a qualitative paper describing the patient-centered development [21]. Effects of the RCT were still pending at the time of writing. Furthermore, within this study, there were 2 arms, namely the guided ACT group and the WLC group. The only distinction between the guided and unguided groups was the provision of guidance through therapeutic email communication. Despite this, the content of the intervention administered to both groups was identical, which is why both groups were examined in this study. Participants could engage with the intervention autonomously by navigating through it individually. The guidance provided was minimal and had a low threshold, consisting solely of an asynchronous chat function.

**Table 2.** Overview of Embrace Pain.

Intervention	Details
Aim	Learning patients to manage their symptoms and pursue psychological flexibility
Duration	8 weeks
Modules	6 (1: “Chronic neuropathic pain”, 2: “On the way to values”, 3: “Away from my values”, 4: “On the road with skills”, 5: “Taking a new road”, 6: “On the road to values: from day to day”)
Investment	2 hours per week
Content	Theory, exercises, mindfulness, metaphors
Multimedia	Text, illustrations, audio files
Groups	ACT^a^: Embrace Pain, including asynchronous therapeutic email guidance WLC^b^: Embrace Pain after 5 months, excluding asynchronous therapeutic email guidance
Software	Karify/Avinty (ISO 27001 and NEN 7510 certified)

a ACT: acceptance and commitment therapy.

b WLC: waiting list condition.

### Recruitment

Participants were selected from the group of participants in the RCT of this project, which was conducted between December 2021 and July 2024 [22]. Inclusion and exclusion criteria are described in the protocol paper previously published [22]. Inclusion criteria regarded being 18 years or older, being identified as having painful sensations by a clinician or self (ie, aching, burning, “pins-and-needles,” shock-like, painful tingling, numbness, and cramps), and experiencing sensations for at least 3 months. Furthermore, a score of 3 or higher on an 11-point pain intensity scale (numeric rating scale), the pain was not present before the administration of chemotherapy, chemotherapy concluded at least 6 months ago, and a score of 1 or higher on the European Organisation for Research and Treatment of Cancer QLQ-CIPN20 Questionnaire was required. The RCT included a total of 112 participants, of whom 25 were invited to participate in an interview. In a previously administered questionnaire within the RCT, patients indicated their willingness to participate in an interview at a later stage. Those who responded affirmatively were considered potential interview participants. Potential interview participants were approached via email within a month after completion of the intervention. Not all patients who expressed willingness in the questionnaire were invited to participate in an interview, because recruitment was stopped when data saturation was reached. During participant selection, efforts were made to ensure a balanced distribution between the guided and unguided groups, as well as gender distribution. It was decided to include both guided and unguided participants, as it was anticipated that the strengths and areas for improvement of the Embrace Pain intervention would exhibit significant overlap between both groups. Efforts were also made to include participants with different levels of engagement, attempting to include both patients with high and low engagement levels. This was estimated by one of the researchers (DvdG), who was aware of the patients’ usage by accessing user data in the intervention’s online system. Excluded were patients who had not activated their account of the intervention Embrace Pain and who were unable or unwilling to participate in an online video call via Microsoft Teams. Patients were able to participate regardless of the time elapsed since they had completed the intervention. Participants sometimes had previous contact via email or phone regarding their participation in the RCT. Some participants had participated in a previous interview with the same researcher (DvdG).

### Development of Interview Guide

The interview scheme was based on 2 qualitative studies into subjective cancer patients’ experiences of online self-management interventions [16,17]. It is important to note that these studies followed the definition of adherence, while the operationalization of these studies fits the concept of engagement and of the subjective experience of use as defined in our study. Furthermore, the input from previous patient interviews regarding user needs in the context of this online intervention [21] was used as input for the interview scheme. The final semistructured interview scheme is presented in Table 3.

**Table 3.** Semistructured interview scheme.

Category	Questions
Motivation to participate	Why did you decide to participate? Did you have any doubts about participating? Why (not)?
Reason for nonusage	Were you able to complete the training? If not, why did you quit the training? What influenced you to quit training?
Experiences with intervention content	To what extent did you find the training relevant and of decent quality? How was your experience with the (1) information sections? (2) exercises? (3) quotes? (4) audio fragments? (5) time investment? (6) mental workload? Were there components that made you want to put more or less time and energy into the training? If so, which ones? Which parts did you find difficult and why?
Experiences with intervention technology	What do you think of the online format of the training? To what extent did you experience problems using a computer, tablet, or phone? To what extent did you experience problems using the online environment?
Experiences with supervisor (if applicable)	What was your experience with the guidance you received during the training?
Personal circumstances	What personal circumstances may have increased your use of the training? Were there any circumstances in your personal life that made it difficult to attend the training? How did you deal with these?
Experiences of intervention effects	Do you feel the training was worthwhile? Why (yes or no)? How did the training change your thoughts and behaviors? Did the training help you to cope better with the neuropathy? Why (not)?

### Data Collection

Interviews were conducted by 1 interviewer with each participant. Interviews were conducted by one of the authors (DvdG) between September 21, 2022, and December 19, 2023. Interviews took place via video calling through Teams. Recruitment for interviews stopped when data saturation was reached [28], indicating that additional interviews yielded no novel information, with recurring topics consistently emerging.

### Data Analysis

Thematic analysis was used for analyzing the data [29]. Audio of the interviews was recorded and transcribed verbatim by 1 author (DvdG) and by a Psychology postgraduate student from Tilburg University. These students contributed to the research study in the context of their data collection task as part of their master’s program. They were mentored by FM or HT in writing their thesis on a related topic and did not contribute to the development of this manuscript. Transcripts were not returned to participants for comment and correction because of the potential burden on patients. DvdG reviewed the transcriptions to confirm accuracy.

All transcripts were inductively coded, deriving themes directly from the data. Text fragments were coded when they involved barriers or facilitators in the use of the online training Embrace Pain from the patient’s perception. When patients discussed potential barriers and facilitators relevant to other patients but not applicable to their situation, these were not coded. The analysis began with open coding, where the primary themes of facilitators and barriers guided the coding process. Once all transcripts were coded, an initial codebook code was reviewed and adjusted if necessary. Axial coding was then applied to explore how the codes related to the themes’ facilitators and barriers. This process was repeated once for verification. Subsequently, the frequency with which each code was mentioned, as well as the number of participants who mentioned each code, was calculated. Ultimately, condensed meaning units were generated for every text fragment that was coded, capturing the core message of each quote. This approach facilitated a comprehensive understanding of the data, organized around the identified themes.

Interviews were coded in Atlas.ti 23 (Lumivero). One transcript was coded by DvdG and FM independently. Differences in coding were thoroughly discussed to ensure consistency and accuracy. Following these discussions, a consensus was reached regarding the appropriate coding approach. Subsequently, DvdG undertook the coding of the remaining transcript, which was performed inductively as well. In cases where uncertainties arose, these were addressed through further discussion to ensure clarity and agreement on the coding process. Due to the differences in background and expertise of DvdG and FM, these discussions were conducted in a manner that allowed for as objective an evaluation as possible.

### Ethical Considerations

This study was approved by the Medical Research Ethics Committee Brabant, the Netherlands (#NL78436.028.21) and the Ethical Review Board of Tilburg University (School of Social and Behavioral Sciences), the Netherlands (#TSB_RP70). Participants signed informed consent upon enrollment in the RCT that included the interview component. Participants did not receive financial compensation for participating in either the interview or the RCT. Only anonymized data were used in the study. Each participant was given a participant number. This number was kept in a secure password-protected document along with the necessary personal information (name, date of birth, email address, date, and time of interview). Only DvdG had access to this file. For all elements of subsequent procedures, including audio files, transcription, and coding, participant numbers were used. This means that no personal information was available in the transcription and analysis process.

## Results

In total, 13 patients participated. However, 1 participant was excluded because of cognitive problems. This eventually resulted in 12 participants, of which 67% (n=8) were female and 33% (n=4) were male. Of these participants, 58% (n=7) were part of the guided ACT group, while 42% (n=5) were part of the WLC and therefore did not receive guidance. In total, 9.3% (n=112) of the participants from the RCT took part in this qualitative study. Participant characteristics are provided in Table 4. Characteristics regarding intervention and usage are presented in Table 5. Interviews lasted 20-30 minutes.

Results can be divided into 2 overarching themes: barriers and facilitators to engagement. Furthermore, 7 codes emerged from the data for barriers and 10 codes for facilitators. An overview of all strategies is presented in Table 6. While the exact numerical values are not the main focus, the table illustrates the facilitators and barriers that were more or less commonly experienced by participants.

**Table 4.** Participant characteristics.

Participant	Sex	Age^a^ (y)	Educational level^b^	Employment status	NRS^c^ (average) at baseline^d^	NRS (worst) at baseline^e^	Type of cancer	Comorbidities	Other long-term consequences of cancer (treatment)
P1	Male	70‐75	High	Retired	7	8	Bladder cancer	High blood pressure	Fatigue, memory difficulties
P2	Female	55‐60	High	Work disability	5	7	Breast cancer	—^f^	Heart problems due to treatment
P3	Female	60‐65	High	Employed	6	8	Multiple myeloma	—	
P4	Male	60‐65	High	Work disability	8	8	Breast cancer	—	Fatigue, reduced physical condition, memory difficulties, concentration difficulties, hormonal problems, stress, anxiety problems, heart problems due to treatment
P5	Female	50‐55	Middle	Unemployed	5	8	Breast cancer, ovarian cancer	Osteoarthritis	Concentration difficulties, memory difficulties
P6	Female	70‐75	High	Employed	7	8	Breast cancer	—	High blood pressure, fatigue, reduced physical condition, memory difficulties, problems with eating or body weight, hormonal problems
P7	Male	60‐65	High	Unemployed	6	8	Multiple myeloma	Heart condition, back pain	Fatigue, reduced physical condition, sleep problems, sexual problems, concentration difficulties, osteoporosis, heart problems due to treatment
P8	Male	65‐70	Middle	Unemployed	4	8	Multiple myeloma	—	
P9	Female	70‐75	Middle	Employed	3	5	Breast cancer	Osteoarthritis	Fatigue, reduced physical condition, hormonal problems, stress
P10	Female	55‐60	High	Employed	3	5	Lung cancer	High blood pressure, depression	Reduced physical condition, sleep problems, sexual problems, concentration difficulties, memory difficulties, depressive symptoms, anxiety symptoms, osteoporosis,
P11	Female	60‐65	High	Employed	4	7	Breast cancer	Osteoarthritis	Concentration difficulties, memory difficulties
P12	Female	55‐60	High	Unemployed	7	8	Colorectal cancer	—	Concentration difficulties, memory difficulties, problems with eating or body weight

a Ranges are shown to ensure anonymity.

b Middle: secondary education or vocational education; high: Bachelor’s degree or higher.

c NRS: numerical rating score.

d Numeric Rating Score (average): “How severe was your pain (on average) in the past week (7 d)?” (0=no pain, 10=the worst pain possible).

e Numeric Rating Score (average): “How severe was your pain at the worst times in the past week (7d)?” (0=no pain, 10=the worst pain possible).

f Not applicable.

**Table 5.** Intervention and usage characteristics.

Participant	Time since the end of intervention	Indicated average time spent on intervention per week	Guidance^a^	Indicated intervention completion (modules completed)
P1	16 weeks	14 hours	Yes	Yes
P2	<1 week	2 hours	No	Yes
P3	7 weeks	8 hours	Yes	Yes
P4	11 weeks	<1 hour	Yes	Yes
P5	20 weeks	2 hours	No	Yes
P6	6 weeks	1 hour	Yes	Yes
P7	10 weeks	1 hour	Yes	Yes
P8	8 weeks	2 hours	Yes	Yes
P9	10 weeks	3 hours	Yes	Yes
P10	3 weeks	—^b^	No	Yes
P11	4 weeks	2 hours	No	Yes
P12	4 weeks	1 hour	No	No (5 out of 6)

a Participants who received guidance were in the guided acceptance and commitment therapy group. Participants who did not receive guidance were in the waiting list control group.

b Not available.

**Table 6.** Barriers and facilitators to engagement.

Theme and code	Number of times coded^a^ (n)	Number of participants^b^ (n)
Barriers		
Program schedule	19	7
Burden	11	7
Lack of guidance	9	6
Irrelevance	7	6
Mindfulness exercises	6	4
Usability	4	4
Missing content	2	2
Facilitators		
Usability	14	12
Recognition	15	10
Positive self-management	18	9
Program schedule	14	8
Symptom management	10	8
Relevance	9	7
Guidance	9	6
Experiential exercises	7	6
Mindfulness exercises	7	5
Value-based living	5	5

a Number of times coded: the frequency with which the code was applied across the various transcripts.

b Number of participants: the number of transcripts, corresponding to individual participants, in which the code was applied.

### Barriers

The most common barrier identified was the program schedule. Many patients indicated having insufficient time for completion:

*I did find 8 weeks of training too tight. … Especially with the effects of cancer, you do have days when you feel bad. And now you had to keep going because of the time frame*.

[P2]

Furthermore, many patients indicated that the timing was unfavorable due to personal schedule or holidays.

*I suddenly had to start, but you have to be able to plan ahead. By then a week had passed. That made me feel rushed*.

[P3]

*We spent about three weeks on vacation during that period. That also takes you out of it and makes it harder to get going again*.

[P12]

Another barrier mentioned by several patients was the overall burden of the intervention. Some patients indicated that it was confronting to work on the training:

*You do also get into a deeper layer. I had a bit of a problem with that, too. … I became aware of something of which I thought ’oh I left that behind me a long time ago’*.

[P3]

Also, some patients indicated physical limitations or cognitive limitations due to chemo brain:

*Because I have some cognitive impairments, processing is difficult. I understand it, but I also forget it very quickly, so I have to go back often*.

[P10]

Burden is also related to remembering to attend the training during the intervention period, as indicated by some patients:

*At the end I ran out of time, also a little bit due to my chemo brain. Then I forget I’m doing that training*.

[P11]

Many patients indicated barriers related to guidance. Several patients who did not receive guidance expressed a need for some form of guidance to be able to ask questions:

*Sometimes I had questions and then I couldn’t ask those questions. … Sometimes I found that quite difficult*.

[P5]

Some other patients indicated that they would have preferred guidance for motivation or reminders:

*It would have helped if once a week or so there was an email with: ‘Is it all working out? Do you have any questions? This is the end date when you should be finished.*’

[P11]

Furthermore, 1 patient who did receive guidance indicated that the delivery of guidance (ie, by email) was not as required:

*...ie, enjoy talking [verbal communication] to someone and then you also get more motivation from them. Like: the coach comes into play, and I have to do well*.

[P6]

Furthermore, several patients indicated that irrelevant content was a barrier. This meant that some patients experienced no connection to their own situation:

*What I always find difficult is when it comes to your partner. I am a mother with adult children who is divorced and who lives alone. I can’t do anything with examples related to partners*.

[P2]

In addition, some patients indicated that they were already familiar with the content:

*Well I have found that I do a lot of things already. A lot of things were not new to me*.

[P5]

Several patients reported experiencing barriers related to the included mindfulness exercises. This relates to the length (P7) or quantity (P9) of mindfulness.

*All those mindfulness exercises, I did that occasionally but most of it I didn’t do. Certainly not the longer exercises*.

[P7]

*I think the training is put together well, but that meditation thing maybe there shouldn’t be too much emphasis on that. It does make sense, but not two of those assignments*.

[P9]

Some patients reported experiencing barriers regarding usability. Some patients indicated difficulties navigating through the training:

*At one point it said, ‘look back at how that went’. Then I think ‘yeah I’m not going to look back’*.

[P9]

Also, one patient indicated that the lack of face-to-face communication was a barrier:

*The emotion in the communication is not there*.

[P1]

Furthermore, one patient indicated problems logging in:

*There were a few email addresses and then I didn’t know exactly how to get to the right address to log in*.

[P7]

Finally, barriers were reported by a few patients due to missing content. One patient indicated that the exercises contained insufficient explanation and examples:

*I found the exercise about the areas of life difficult. … Maybe it would be good to mention in the first exercise ‘if you don’t get there, you can still do the alternative exercise.’*

[P3]

Another patient indicated that they missed the opportunity for peer contact:

*I would like to hear other people’s response. … I think if you are in a group where you also hear what the others are saying, you can have a lot of support from that*.

[P5]

### Facilitators

A facilitator that many patients mentioned was usability. Several patients indicated benefiting from the flexibility of the training, allowing them to use it at their convenience, pausing when needed:

*It was nice. Sometimes on the tablet, sometimes briefly on the phone. You can put it away again for a while.*

[P12]

Several patients also indicated that the freedom in the training facilitated their use:

*I could look back. I did that regularly. Then I looked back a few sessions to continue*.

[P8]

Also, a few patients indicated that the training was easy to use:

*I found the course very doable online. It was clear and straightforward. The structure was consistent, and I could find everything very easily*.

[P2]

Furthermore, many patients indicated recognition as a facilitator. Several patients indicated that they found recognition in the fictitious personas:

*I am reminded of the example given in training of a tennis player who had back pain. If he played tennis hard, he would forget the pain. I feel the same way*.

[P1]

Several patients also indicated that the examples and personas clarified the information and theory that was provided:

*When you see practical examples like this, it also indicates how differently you can deal with it. That supports the theory. I really liked that combination*.

[P11]

In addition, many patients indicated facilitators related to positive self-management. For several patients, the training was a way to find reflection:

*Well, for me, the most important thing was the insight that I put a lot of limitations on myself. I knew that, but it went much further than I actually realized*.

[P10]

Also, several patients indicated that they appreciated being able to further develop themselves through the training:

*Here you learned about negative thoughts and what can help you turn that around. I also found that helpful*.

[P3]

Furthermore, some patients indicated that they received confirmation of their coping through the training:

*A lot of things I was already doing. ... It was often just confirmation for me.*

[P5]

Many patients indicated the program schedule as a facilitator, which was mainly related to the flexibility of the program schedule. This is mainly related to being able to work on the training whenever and wherever the patient wants:

*You can grab it at your own time when you have time for it. You just sit down for it and you don’t have to leave the house*.

[P4]

Some patients appreciated the option to pause or request extensions for the training, particularly during personal circumstances that temporarily hindered use:

*During the training I found out that the cancer is back. Then I took a break from it.*

[P10]

There were also some patients who indicated that they valued being able to make their own choices about completing the exercises:

*In the mindfulness part, you got these 20-minute sessions, and you got a shorter one. Actually, you were supposed to apply both, but then I backed off. ...Then I chose to do the shorter one*.

[P9]

Several patients indicated symptom management as a facilitator. Several patients felt that participation in training might improve their symptoms:

*I’ve tried quite a bit to combat neuropathy and anything I come across is welcome to me.*

[P1]

Furthermore, some patients indicated that they liked getting tips:

*I had gotten tips from you guys that I could just apply to that as well. That generally works pretty well I must say.*

[P12]

There were also some patients who experienced psychoeducation as a facilitator:

*I often really enjoyed reading about the theory about mechanism of a human being of why we do things and what the effect is if you choose to avoid the pain. I found that very useful to read.*

[P11]

Finally, certain elements in the training were identified as facilitators by several patients. Several patients indicated that the relevance of the training was a facilitator because this made it possible to apply the training to one’s own situation:

*It was relevant because I do have the symptoms. Regardless of where they come from. … The complaints are there, and you only refer to them*.

[P10]

Furthermore, several patients liked the option for guidance for various reasons, such as motivation, clarification of information, and feedback:

*I really liked that I had that online guidance. ... That does keep it going. I knew she [guide] would respond every Friday, and I would fill in some things in advance.*

[P3]

In addition, several patients indicated that they experienced both experiential exercises (P5) and mindfulness exercises (P8) as facilitators.

*Yes, I liked those because then you get to know yourself better and you see what you are doing.*

[P5]

*Especially that 4 min breathing exercise where you focus on breathing in and out and where the air goes. I sometimes did it in bed at night after completion of the training, because the neuropathy is more pronounced then.*

[P8]

Content related to value-based living was also perceived by several patients as a facilitator:

*You never think about that so much. You get sucked into all the things that maybe you don’t feel like doing. This made you look at: ‘What are your values? What do you like to do? What are the things that are important to you?’ That impressed me.*

[P9]

## Discussion

### Principal Findings

This study qualitatively examined barriers and facilitators to engagement of an online self-management intervention for cancer survivors with chronic painful CIPN. Participants generally reported more facilitators than barriers. Facilitators most often related to the perceived ease of use of the intervention, feelings of recognition and validation, the opportunity to actively engage in self- and symptom management, and a favorable program schedule. In contrast, commonly reported barriers included an unfavorable program schedule, perceived burden, lack of guidance, and content irrelevance. Notably, some factors, such as the program schedule, were described both as facilitators and barriers, underscoring the influence of individual preferences and circumstances. Factors seem to include a combination of mainly psychological, intervention-related, and technology-related characteristics. Furthermore, certain aspects are related to illness or individual circumstances. These findings align with previous research on barriers and facilitators [17,18], as well as theoretical frameworks like the conceptualization of engagement in digital behavior change interventions [7,12].

This study revealed that many patients felt a strong sense of recognition in the online intervention, which is likely related to the inclusion of quotes and personas added to make the program more personal, which were based on real patient experiences [21,30,31]. These patient experiences, depicted in a first-person narrative, can contribute to the feeling of social support that can help alleviate feelings of isolation for patients in coping with symptoms [32-34]. However, patient experiences shared by individual users may contain unbalanced information or misinformation [35]. In this online intervention, therefore, researcher-written narratives based on experiences shared by patients were incorporated. This can be seen as an accessible and controlled way of incorporating patient experiences in online interventions to increase the sense of social support.

Furthermore, this study has shown that many patients perceived participating in the online intervention as a pleasant way of engaging in positive self-management. Self-management empowers cancer survivors to enhance their health and well-being [36]. Previous research has shown that theoretically based self-management interventions for various types of chronic diseases, including cancer, can positively influence outcomes such as quality of life, coping, health goals, self-regulatory skills, and self-efficacy [36-39]. Self-efficacy for symptom management in patients with cancer is defined as “the ability to implement behaviors to prevent, recognize, and relieve symptoms” and is a vital component in enabling patients to effectively self-manage their cancer-related symptoms [40,41]. Self-efficacy empowers patients to change their self-management behaviors and helps patients set realistic goals in coping with the symptoms [40,42]. This closely aligns with the acceptance of symptoms and the pursuit of a value-driven life, which reflects the ACT-based approach of the online intervention used in this study. This alignment may help explain why active engagement in positive self-management and symptom management emerged as a frequently reported facilitator in this study.

While for many patients, the flexibility of the training program was a facilitator, many patients experienced the training program as a barrier. In this case, this is related to both unfavorable start time and insufficient time to complete the training due to personal situations, in the context of the study. This aligns with previous studies regarding the use of online psychological interventions, which have shown that time-related factors (eg, not having enough time or being too busy) deteriorate adherence and engagement [15,17,43]. It is important to mention that in this study, the start of the training was not aligned with the participants in advance. In the study, participants received an invitation at the start, and had 8 weeks for completion, which was only extended upon request. Based on this study’s results, it is recommended to initiate online interventions in coordination with participants. In addition, it is suggested that the intervention’s total duration is sufficiently long, incorporating several weeks of flexibility in scheduling (eg, 9 weeks for an online intervention with 6 components). It can also be considered to allow unlimited access to the intervention. We also recommend that participants be encouraged to contact the contact person or supervisor of the online intervention if they encounter problems, so that it can be assessed when an extension of access is needed to avoid dropout.

Furthermore, barriers identified in this study included the burden and chemo-brain that patients may suffer from. Chemo-brain is known to be a consequence of chemotherapy, which is characterized by self-perceived thinking and memory problems [44]. In this study, for example, patients reported that the training was too stressful or that they had concentration problems. However, this does not only apply to chemo-brain, but also to other physical and mental symptoms caused by cancer or cancer treatment, which causes people to have more difficulty performing daily activities. These may include fatigue, which increases the need for more frequent breaks, or disease-related fear, which may make individuals more vulnerable and prone to heightened feelings of fear in general. Thereby, having a higher age with additional physical and cognitive challenges, which is often the case in cancer survivors, can make this more difficult. Therefore, a low cognitive effort of the online intervention is necessary for proper understanding of the content and effective use [45]. This can be achieved by dividing the intervention into short, manageable sections to reduce the complexity on the pages and to provide simple navigation between pages and sections [45]. Patients also indicated that they valued the ability to schedule the use of the intervention within the provided time frame of the training themselves, as well as the ability to pause when needed, which has also been shown in previous qualitative research regarding computerized CBT for depression [46]. This is also relevant for personal situations of participants, for example, in case of receiving a new cycle of chemotherapy treatment during the intervention period. During the study, it became evident that not all participants were fully aware of this optional flexibility in the schedule. For instance, 1 participant did not report receiving a new cycle of chemotherapy, which ultimately limited her time to complete the intervention due to restricted access, as a modified schedule had not been discussed. This also applied to, for example, holidays or family issues, which aligns with earlier research [11]. Given the complex circumstances participants often encounter, it is essential, in addition to providing an intervention that considers factors such as breaks and division into manageable sections, to engage in dialogue with users to identify an approach that aligns with their individual needs, ensuring the intervention is as adaptable as possible.

In terms of guidance, patients who had access to it indicated finding it valuable, despite not all of them using it consequently. Among those without access to guidance, some expressed a clear need for it. For example, these patients would have appreciated the opportunity to ask questions for clarification on information or exercises, as well as to discuss personal circumstances that made engaging with the online intervention more challenging. Previous research has shown that online interventions with guidance compared with nonguided versions result in better outcomes in satisfaction, usage, and adherence [19,20,47]. Also, an earlier study examining a self-guided online CBT intervention for patients with CIPN suggests that counseling could be a useful addition [1]. However, a systematic review examining engagement with online psychological interventions found that preferences for guidance varied greatly, ranging from a need for intensive support to enhance engagement to a preference for no guidance to maintain anonymity and freedom [15]. Therefore, offering optional guidance rather than mandatory support appears to be recommended.

### Limitations and Strengths

Some limitations of this study should be mentioned. First, no results were available from patients with very low engagement levels, since these people were reluctant to participate in interviews. Several attempts were made to include these patients, but despite these efforts, they remained unwilling to take part, and some potential respondents did not respond at all. It was not possible to engage in discussions with individuals who were dissatisfied or who had discontinued participation prematurely, suggesting that this group was unwilling to discuss their experiences with us. The sample in this study is therefore not representative of the entire user group. Barriers that might exist with low-engaged patients, such as lack of time and low levels of motivation [15,17], were therefore not completely reflected in this study. Furthermore, people with low levels of use might also comprise patients who realized they were already proficient in managing their symptoms and consequently discontinued the intervention. Unfortunately, obtaining insights into this matter was not possible because these patients did not participate in the interviews. Therefore, the results of this study must be interpreted with caution. Second, there was a lot of difference in the time between intervention completion and the interview. Possibly, patients with a longer period between the intervention and interview may have remembered fewer details of their experience with the intervention, which could have also affected the results of this study. Third, the results of this study may be less applicable to other self-management interventions. Each intervention has its characteristics in terms of tasks, technology, and users, all of which affect ultimate use [7,48-50]. Consequently, the experiences encountered by patients also vary. Fourth, the study sample included highly educated and highly motivated patients who experienced little or no difficulty with language and the use of technology. Therefore, the transferability of the results to groups with lower education levels and lower technology literacy levels might be limited. Fifth, no participant checks were performed because of the possible burden on patients. However, in terms of validating the results, it is desirable to perform participant checking in future studies.

Some strengths should also be emphasized. First, interviews were semistructured, enabling patients to freely express their individual experiences without being constrained by the interview’s format. The core questions were addressed in all interviews, leading to a consistent structure across all interviews. Second, interviews were conducted with patients from all conditions, including both those who received therapeutic email guidance and those who did not. This enabled the assessment of the experienced barriers and facilitators associated with the availability or unavailability of guidance. Third, interviews took place via video call, enabling vulnerable and disabled patients with cancer to participate without being hindered by, for example, physical limitations.

### Conclusions

This study qualitatively identified facilitators and barriers to engagement in an online self-management intervention for cancer survivors with chronic painful CIPN. Facilitators and barriers varied greatly and were dependent on personal situations. Recommendations could be given in terms of recognition, program schedule, burden, and guidance. Consideration of such recommendations following qualitative evaluations is imperative to ensure careful implementation in practice.

## Abbreviations

ACT: acceptance and commitment therapy
CBT: cognitive behavioral therapy
CIPN: chemotherapy-induced peripheral neuropathy
RCT: randomized controlled trial
WLC: waiting list condition
